# Dual Antibody-Conjugated Amyloid Nanorods to Promote Selective Cell–Cell Interactions

**DOI:** 10.1021/acsami.0c21996

**Published:** 2021-03-24

**Authors:** Weiqiang Wang, Marcos Gil-Garcia, Salvador Ventura

**Affiliations:** Institut de Biotecnologia i de Biomedicina and Departament de Bioquímica i Biologia Molecular, Universitat Autònoma de Barcelona, Bellaterra (Barcelona) 08193, Spain

**Keywords:** amyloid, dual- or multitargeting, multivalency, antibody, nanorods, nanomaterials

## Abstract

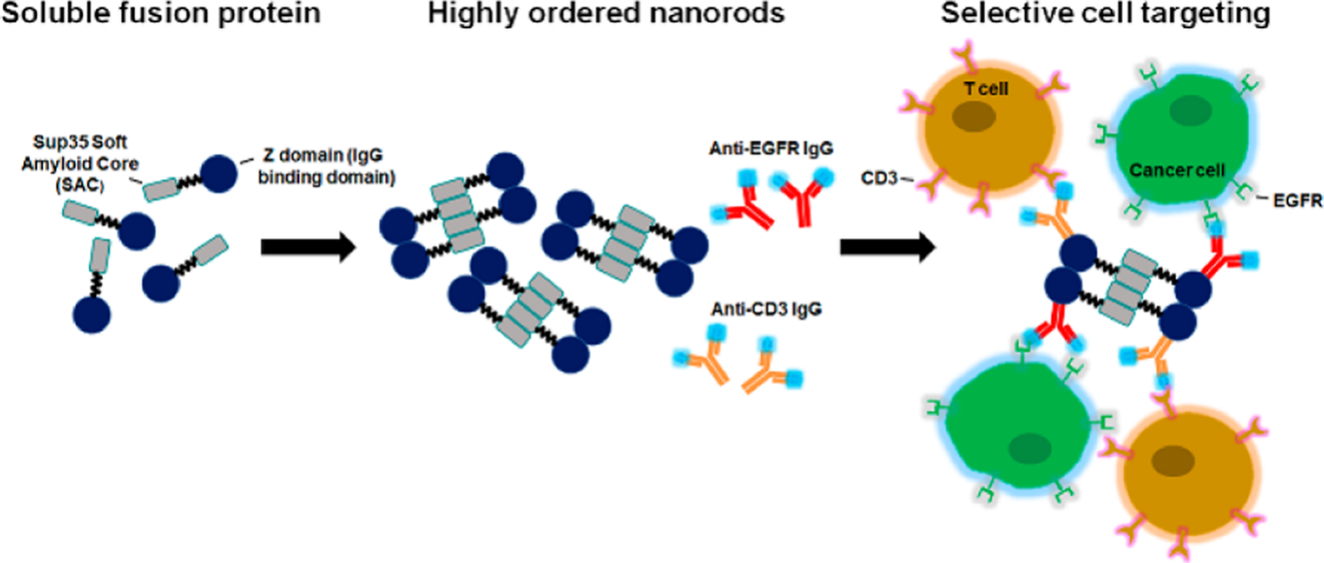

Grafting biomolecules on nanostructures’ surfaces is an increasingly used strategy to target pathogenic cells, with both diagnostic and therapeutic applications. However, nanomaterials monofunctionalized by conjugating a single type of ligand find limited uses in pathologies/therapies that require two or more targets/receptors to be targeted and/or activated with a single molecular entity simultaneously. Therefore, multivalent nanomaterials for dual- or multitargeting are attracting significant interest. This study provides a proof of concept of such nanostructures. We have recently developed a modular methodology that allows obtaining amyloid-based materials decorated with active globular domains. Here, this approach is exploited to generate functional amyloid fibrils displaying antibody capture moieties. A high antibody binding affinity and capacity for the resulting nanofibrils, whose size can be manipulated to obtain homogeneous nanorods with high biocompatibility, are demonstrated. These nanorods are then used for specific antibody-mediated targeting of different cell types. Simultaneous conjugation of these nanorods with different antibodies allows obtaining a mimic of a bispecific antibody that redirects T lymphocytes to tumoral cells, holding high potential for immunotherapy. Overall, the work illustrates a modular and straightforward strategy to obtain preparative quantities of multivalent antibody-functionalized nanomaterials with multitargeting properties without the need for covalent modification.

## Introduction

Tunable nanomaterials with large surface/volume ratios and multiple functional groups are emerging as novel platforms for diagnosing and treating diseases.^1^ In comparison with small molecules, these materials, including nanotubes, micelles, and protein–polymer conjugates, exhibit favorable pharmacokinetics^2^ since they can accumulate at higher concentrations and for a longer time at pathological sites, an effect named as enhanced permeability and retention.^3^ Moreover, their superficial functional groups permit the grafting of tailored biomolecules.^4^ The incorporation of specific ligands that target pathogenic cells is expected to minimize the materials’ toxic side effects and improve their therapeutic efficacy by selective targeting. So far, most of the efforts have been focused on synthetic monofunctional nanomaterials with a single type of ligand intended for a specific target, such as RGD peptides,^5^ monoclonal antibodies,^6^ and other proteins.^7^ However, many diseases are multifactorial, and monospecific conjugates display low effectiveness for their treatment.^8^ In these occasions, multivalency is a requirement, and two or more targets/receptors should be targeted and eventually activated with a single molecular entity.^9,10^

The concept of dual targeting was initially implemented in the creation of bispecific antibodies (BsAbs), in which each of the two different variable regions targets a distinct antigen or epitope. This allows the simultaneous inhibition of two cell surface receptors and enhances agonism through receptor clustering, blocking two ligands, or recruiting T cells to cancer cells,^11^ resulting in a highly increased targeting and therapeutic efficacy.^12^ However, issues such as low yields,^13^ molecular heterogeneity,^14^ short half-time *in vivo*,^15^ and toxic side effects^16,17^ have limited the clinical applications of BsAbs. Dual-targeting nanoparticles, conjugating two different small molecules,^18^ peptides,^1^ monoclonal antibodies,^19^ or binding proteins,^20^ are being developed to overcome BsAb limitations and extend their applications.

The discovery of functional amyloids^21^ has inspired the building up of functionalized amyloid-based nanomaterials.^22^ These bioactive, biodegradable, and biocompatible peptide or protein-based nanomaterials have been used for biological and biomedical applications, ranging from cancer therapy, bioimaging, or tissue engineering to regenerative medicine.^23^ Self-assembled peptide-based nanomaterials offer a high surface area versus the volume ratio and constitute stable superstructures with suitable pharmacokinetics.^24^ Nanomaterials are usually synthesized with a series of complex processes, in which the amyloid scaffold is formed first, and the ligand is covalently conjugated afterward, but this implies a significant inactivation, especially if the ligand has a proteic nature.^25^ Indeed, the major advantage of protein-based materials is the ability to modify their functionalities by simple genetic redesign, as long as the globular domains remain folded and active in the assembled state.

Recently, we have been successful in the design of highly ordered amyloid-like nanofibrils containing well-folded and highly functional proteins using a modular approach. In particular, a soft amyloid core (SAC) of the Sup35 yeast prion was used as the driving force for self-assembly, and it was fused using a flexible linker to a globular domain. The fusion protein is produced in a soluble form at high yield, but it can be induced to form a fibrillar structure, sustained by the Sup35-SAC spine, to which the globular-and-folded domains are attached.^26^ Thus, the appended globular protein remains bioactive and accessible. A similar approach has been applied to manufacture functionalized nanofibrils decorated with the Z-domain,^27^ a designed analogue of the B domain from *Staphylococcus aureus* protein A that binds with high affinity to antibodies.^28,29^ It was rationalized that the Z-domain fusion to Sup35-SAC might constitute an optimal strategy to produce amyloid fibrils for dual targeting. Following this hypothesis, we obtained biocompatible antibody-decorated multivalent nanorods that can recognize and promote interactions between different cell types, such as T lymphocytes and tumoral cells.

## Results and Discussion

### Design of a Fusion Protein to Build Up Antibody Capturing Nanofibrils

In order to create functional amyloid fibers with antibody capturing activity, Sup35-SAC was fused to the Z-domain^27^ (Figure S1).^28^ The Z-domain is a 58 residue-long protein (6.6 kDa) whose three-dimensional structure consists of a bundle-like structure composed of three α-helices. In contrast with the larger green fluorescent protein (GFP) and carbonic anhydrase proteins, which required a separation from Sup35 SAC of at least eight residues to form ordered fibrils,^26^ molecular modeling^30^ suggested a five-residue flexible linker (SGSGS) should suffice to allow amyloid fibril formation without significant steric constraints for the Z-domain. This will reduce the entropic cost of immobilizing the initially disordered N-terminus of the protein fusion into a rigid amyloid structure. The Z-domain is able to bind the Fc region of antibodies independently of their origin and subclass with high affinity. If the protein fusion (Sup35-Z) self-assembles into amyloid nanostructures, such assemblies can be decorated with any desired antibody.

### Sup35-SAC Does Not Alter the Solubility, Conformation, Thermodynamic Stability, and Affinity of the Appended Z-Domain

In order to use Sup35-Z for building an antibody-capturing nanomaterial, the N-terminal Sup35-SAC must not modify the solubility, native structure, and stability of the adjacent Z-domain, and therefore, it does not affect the potential antibody binding activity of the fusion.

We expressed the Sup35-Z fusion protein (10 kDa) in *Escherichia coli*. The protein was localized entirely in the soluble fraction, from where it was purified at a high yield (62 mg/L) (Figure S2A). Then, we compared the secondary structure content of purified Sup35-Z and the Z-domain alone (Z-domain) at pH 7.4 and 25 °C by analyzing their far-UV circular dichroism (CD) spectra. The two proteins’ spectra are very similar and display the typical α-helical signals (Figure S2B). The thermal unfolding of both proteins at pH 7.4 and 25 °C was followed by monitoring the variations in ellipticity at 222 nm, which reports on the stability of the Z-domain α-helical structure (Figure S2C). The obtained melting curves were similar, with a single cooperative transition being observed, consistent with a two-state unfolding reaction. In both cases, the Z-domain was highly stable and not completely denatured, even at 90 °C. Fitting of the data to a two-state reaction rendered apparent melting temperatures of 74.7 ± 0.8 °C and 74.5 ± 1.0 °C for Sup35-Z and the Z-domain alone, respectively. All these data indicate that, as intended, the Sup35 SAC does not impact the solubility, structure, and stability of the adjacent globular domain, consistent with our previous studies on other protein folds.^26^

However, it should also be discarded that the N-terminal exogenous sequence’s presence causes steric impediments for antibody binding to the Z-domain or hides the antibody binding site. To exclude these possibilities, we used soluble Sup35-Z and the Z-domain to purify IgG antibodies from a complex matrix, such as bovine blood serum. The proteins were immobilized in NI-NTA columns through their respective His6 tags, and the serum was chromatographed. The identity of the purified proteins was analyzed by sodium dodecyl sulfate-polyacrylamide gel electrophoresis (SDS-PAGE), which, for both proteins, revealed the presence of three major bands at ∼75, ∼50, and ∼25 kDa, corresponding to IgGs and their heavy and light chains, respectively, without unspecific binding to highly abundant serum proteins, such as serum albumin (Figure S3). The data suggest that soluble Sup35-Z captures IgGs from serum with an efficiency comparable to that of the Z-domain.

### Sup35-SAC Induces the Formation of Sup35-Z Amyloid Fibrils

**Figure 1:**
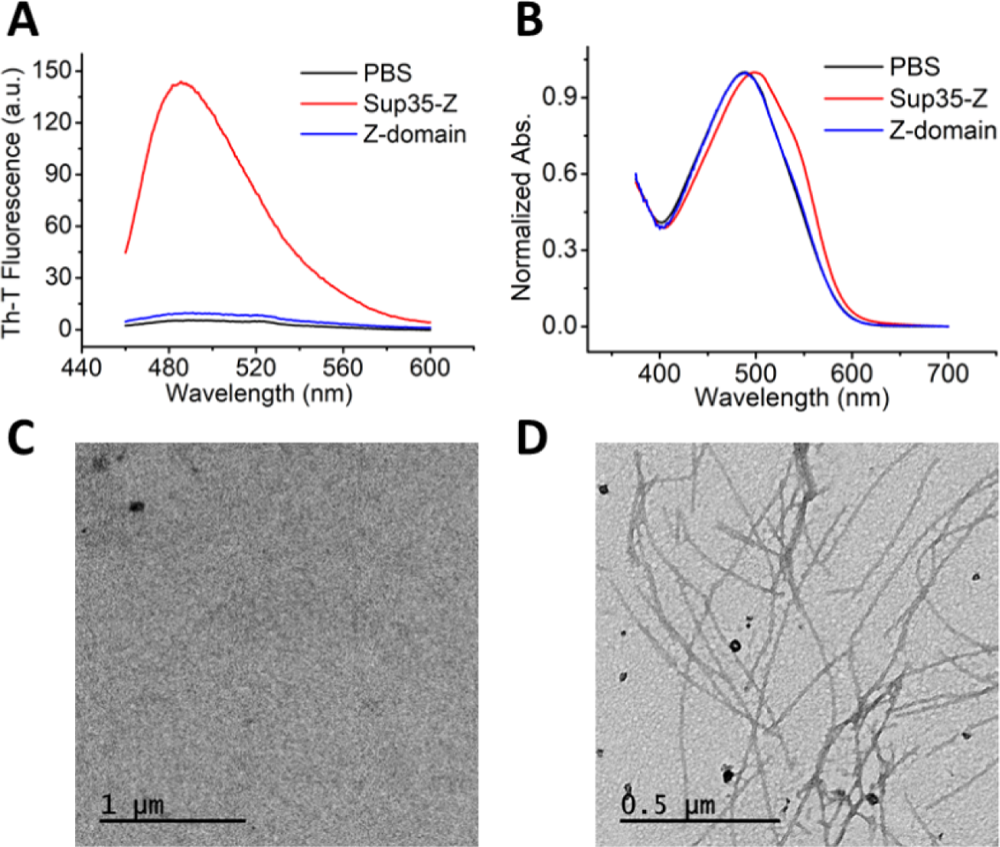
Biophysical characterization of Sup35-Z fibrils. Sup35-Z and Z-domain proteins were incubated for 5 days and analyzed by measuring (A) Th-T fluorescence emission and (B) CR absorbance. Z-domain and Sup35-Z are shown in blue and red, respectively. Phosphate-buffered saline (PBS) alone was included as a control (black line). Representative TEM images of incubated proteins upon negative staining: (C) Z-domain and (D) Sup35-Z. The scale bar represents 1 μm and 0.5 μm, respectively.

The amyloid-specific dyes Thioflavin-T (Th-T) and Congo red (CR) were used to evaluate if the Sup35-Z protein fusion self-assembles into amyloid-like structures under native conditions. Sup35-Z and Z-domain were incubated at pH 7.4 and 37 °C for 5 days. Th-T is a dye in which fluorescence emission maximum at 488 nm is increased when incubated with amyloid structures.^31^ The Th-T fluorescence emission signal was largely increased in the presence of Sup35-Z, whereas the Z-domain alone presented a negligible effect (Figure 1A). In this way, CR binding was observed for Sup35-Z, resulting in a red shift of the CR absorption spectrum, characteristic of an amyloid structure,^32^ whereas the Z-domain did not promote such spectral shift (Figure 1B). The morphological analysis of the two protein solutions by negative staining and transmission electron microscopy (TEM) further corroborated the presence of typical long and unbranched amyloid fibrils of 12.7 ± 0.7 nm width for Sup35-Z (Figure 1D). In contrast, the Z-domain solution did not display any detectable ordered aggregates (Figure 1C).

To assess if, as previously described for other protein folds,^26^ the Z-domain remained folded in the Sup35-Z assembled state, attenuated total reflectance Fourier transform infrared spectroscopy (ATR–FTIR) was used to characterize the secondary structure content of Sup35-Z amyloid fibrils. The Z-domain’s all-alpha fold should allow us to track its native state when embedded in the amyloid fibrillar structure, known to be β-sheet-enriched. We recorded the fibrils’ infrared spectra in the amide I region of the spectrum (1700–1600 cm^–1^), corresponding to the absorption of the main chain carbonyl group and dependent on the protein structure. The spectra’s deconvolution allowed identifying the secondary structure components and their relative contribution to the primary signal (Figure S4). The spectra showed two major signals assignable to the contribution of intermolecular β-sheets (1626 cm^–1^) and α-helices (1654 cm^–1^), accounting for 44.0 and 52.6% of the spectral area, respectively (Table S1). The first signal likely arises from the Sup35-SAC amyloid spine and helical Z-domains, respectively.

All these data indicated that Sup35-SAC is both necessary and sufficient to promote the self-assembly of the Sup35-Z fusion into amyloid-like fibers, where the Z-domain remains in a folded conformation.

### Antibody Binding Capacity of Sup35-Z Amyloid Fibrils

**Figure 2:**
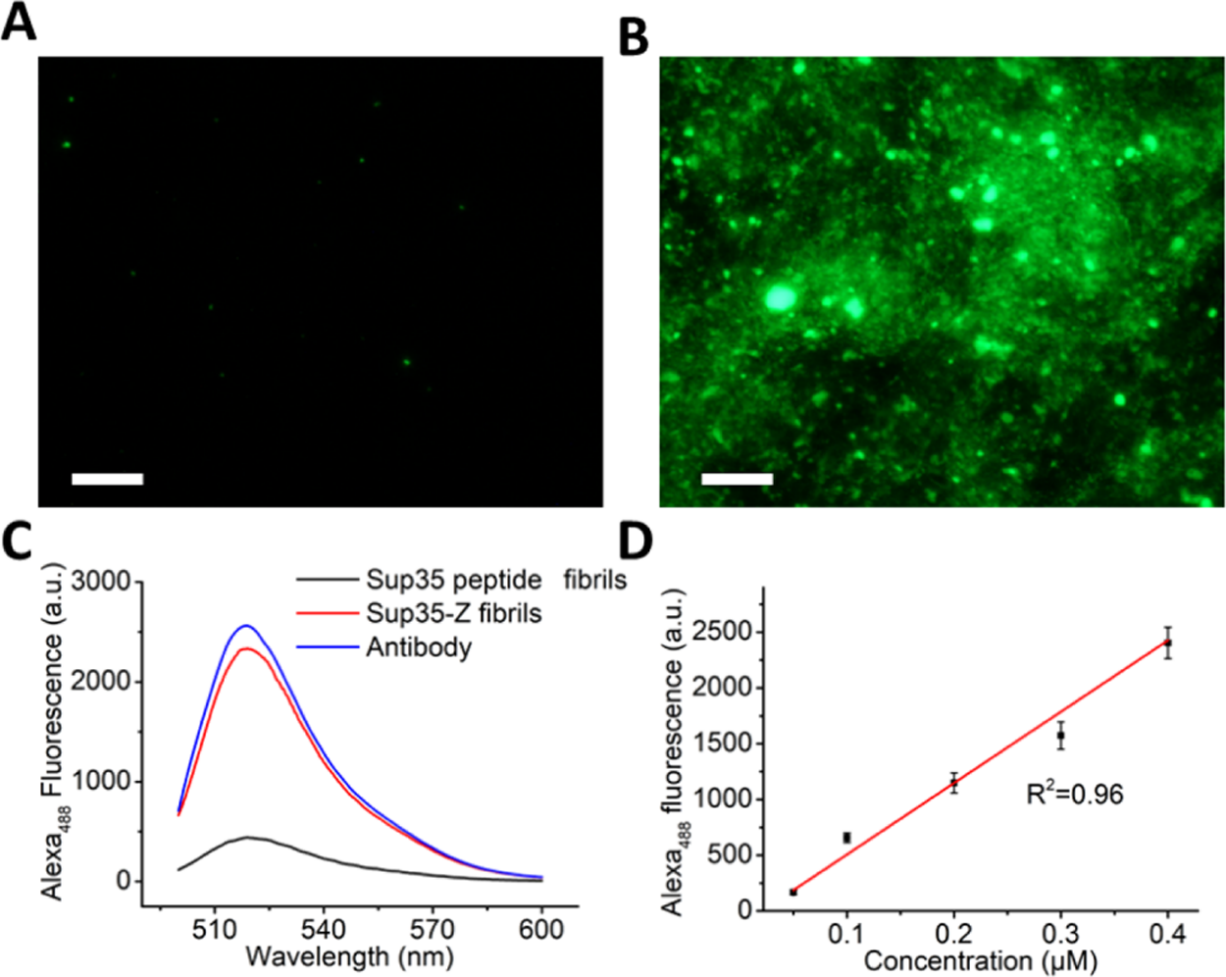
Antibody binding affinity of Sup35-Z fibrils. Representative fluorescence microscopy image of fibrils incubated with single IgG labeled with Alexa 488: (A) Sup35 peptide fibrils and (B) Sup35-Z fibrils. The scale bar represents 50 μm. (C) Fluorescence emission spectra of incubated fibrils at 0.4 μM. The blue line represents the fluorescence spectra of the antibody alone. (D) A linear plot of the fluorescence intensity of incubated fibrils as a function of the fibrils’ concentration.

If, as intended, folded Z-domains are exposed in the periphery of the amyloid fibrils and thus accessible, this would allow obtaining nanostructures decorated with the desired antibody. To confirm that this was the case, we incubated preformed Sup35-Z fibrils and fibrils formed by the Sup35-SAC peptide alone,^33^ with 2 μg of an antibody labeled with Alexa 488 at room temperature for 30 min. Then, the fibrils were harvested and washed three times to remove any unbound antibody and resuspended in PBS. When imaged via fluorescence microscopy, using a fluorescein isothiocyanate (FITC) filter (excitation at 465–495 nm), highly fluorescent aggregates were observed for Sup35-Z fibrils, whereas Sup35-SAC fibrils were devoid of fluorescence (Figure 2A,B). To determine the Z-domain’s antibody capture capacity when embedded in the fibrils, we incubated the green-labeled antibody with fibrils in the range of 0–0.4 μM. Then, the fluorescence emission spectra of the fibrils after precipitation and washing were recorded. Incubated Sup35-Z fibrils exhibited a fluoresce maximum at ∼518 nm, which is also observed in a labeled-antibody solution, whereas Sup35-SAC fibrils did not exhibit any significant Alexa 488 fluorescence signal (Figure 2C). A titration of the fluorescence of Alexa 488 as a function of incubated fibrils indicated that Sup35-Z fibrils have a binding capacity of ∼2.5 μg IgG per μg of fibrils (Figure 2D).

To further confirm the antibody binding affinity of Sup35-Z fibrils in a complex matrix, we incubated the fibrils with bovine blood serum for 30 min, followed by precipitation and washing steps and elution of the fibril-bound protein with glycine-HCl buffer pH 3.0. The analysis by SDS-PAGE confirmed that the Sup35-Z fibrils bind preferentially to antibodies, as evidenced by the heavy- and light-chain bands in the gel, with little contamination of abundant proteins such as serum albumin (Figure S5). To assess if Sup35-Z fibrils are stable under physiological conditions, a requirement for biomedical applications, we incubated Sup35-Z fibrils in bovine blood serum for up to 3 days. SDS-PAGE analysis indicated that the fibrils are stable and not degraded under these conditions (Figure S6).

Overall, the data in this section indicate that Sup35-Z fibrils display a remarkable antibody capturing activity in both defined and complex media and that this property is not due to unspecific binding to the amyloid macromolecular structure but due to the folded Z-domains in the fibrils.

### Accessibility and Functionality of the Conjugated Antibody on Sup35-Z Fibrils

Another requirement to build up functional antibody-conjugated nanofibrils is that the antibody displayed in Sup35-Z fibrils keeps its intact structure and can target the desired antigen epitope. To assess if this was the case, we incubated Sup35-Z fibrils with a mouse anti-GFP antibody. These antibody-bound fibrils were then incubated with soluble GFP for 30 min, precipitated, and washed 3 times to eliminate any unbound GFP. As imaged by fluorescence microscopy, the presence of green fluorescent aggregates indicated that the antibody-conjugated fibrils target the intended antigen (Figure S7B), whereas GFP does not bind to Sup35-Z fibrils if they are not previously incubated with the antibody (Figure S7A).

On the other hand, we incubated the mouse anti-GFP antibody bound Sup35-Z fibrils with a goat antimouse antibody labeled with Alexa 555 and measured the resulting fluorescence spectra after fibril precipitation and washing. Sup35-Z fibrils were also incubated directly with the goat antimouse antibody labeled with Alexa 555 and treated in the same way. Fibrils incubated with the primary and goat antimouse antibodies exhibited a much higher fluorescence maximum at ∼570 nm than fibrils incubated with the goat antimouse IgG alone (Figure S7C). This is expected since several copies of goat antimouse secondary antibody can potentially bind to each primary antibody.

Overall, the data indicated that the antibody bound to the fibrils keeps its intact structure, binds its antigen, and can be targeted by a specific secondary antibody, resulting in a significant amplification of the fluorescence signal.

### Dual Antibody Binding to Sup35-Z Nanofibrils

**Figure 3:**
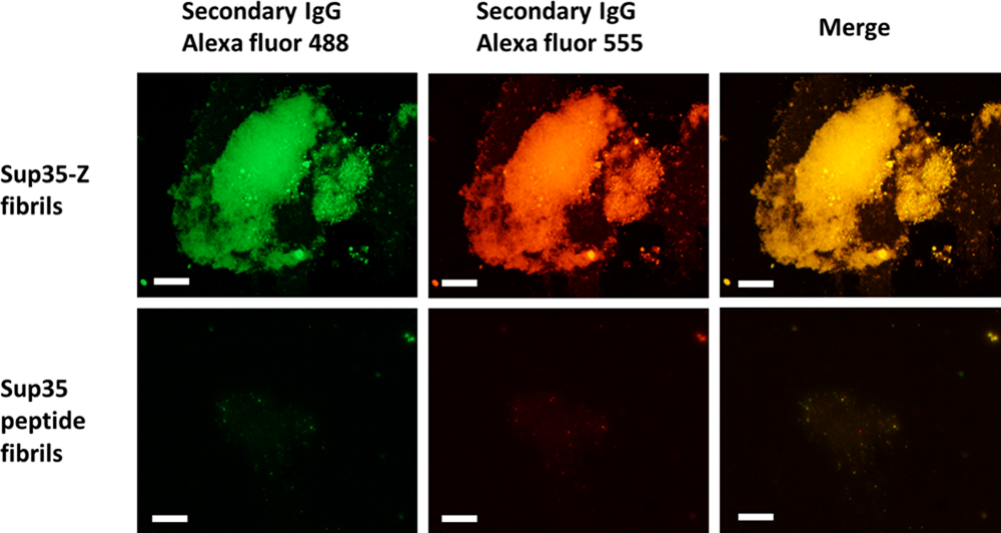
Double antibody binding of Sup35-Z fibrils. The representative fluorescence image of the Sup35-Z fibrils (upper panel) and Sup35 peptide fibrils (bottom panel) incubated simultaneously with two secondary antibodies: rabbit antimouse antibody labeled with Alexa 488, and goat antirabbit antibody labeled with Alexa 555. The scale bar represents 50 μm.

In principle, the Sup35-Z fibrils could be endorsed with multivalency by conjugating them simultaneously with different antibodies. To test if this was possible, the fibrils were incubated simultaneously with two different antibodies labeled either with Alexa 488 or with Alexa 555. After precipitation and washing, the fibrils were imaged using fluorescence microscopy and a FITC filter (excitation at 465–495 nm) or a TxRed filter (excitation at 540–580 nm). The particles were green and red in the corresponding channels, and both signals perfectly overlapped when merged (Figure 3, upper panel). In contrast, Sup35-SAC fibrils incubated with the two antibodies, in the same way, did not exhibit any significant fluorescence (Figure 3, bottom panel). Thus, the data indicate that the Sup35-Z fibrils can be multifunctionalized specifically. Controlling each antibody’s proportion in the initial mixture allows obtaining fibrils decorated with the desired ratio of them (Figure S8).

### Sup35-Z Nanorods Are Biocompatible

**Figure 4:**
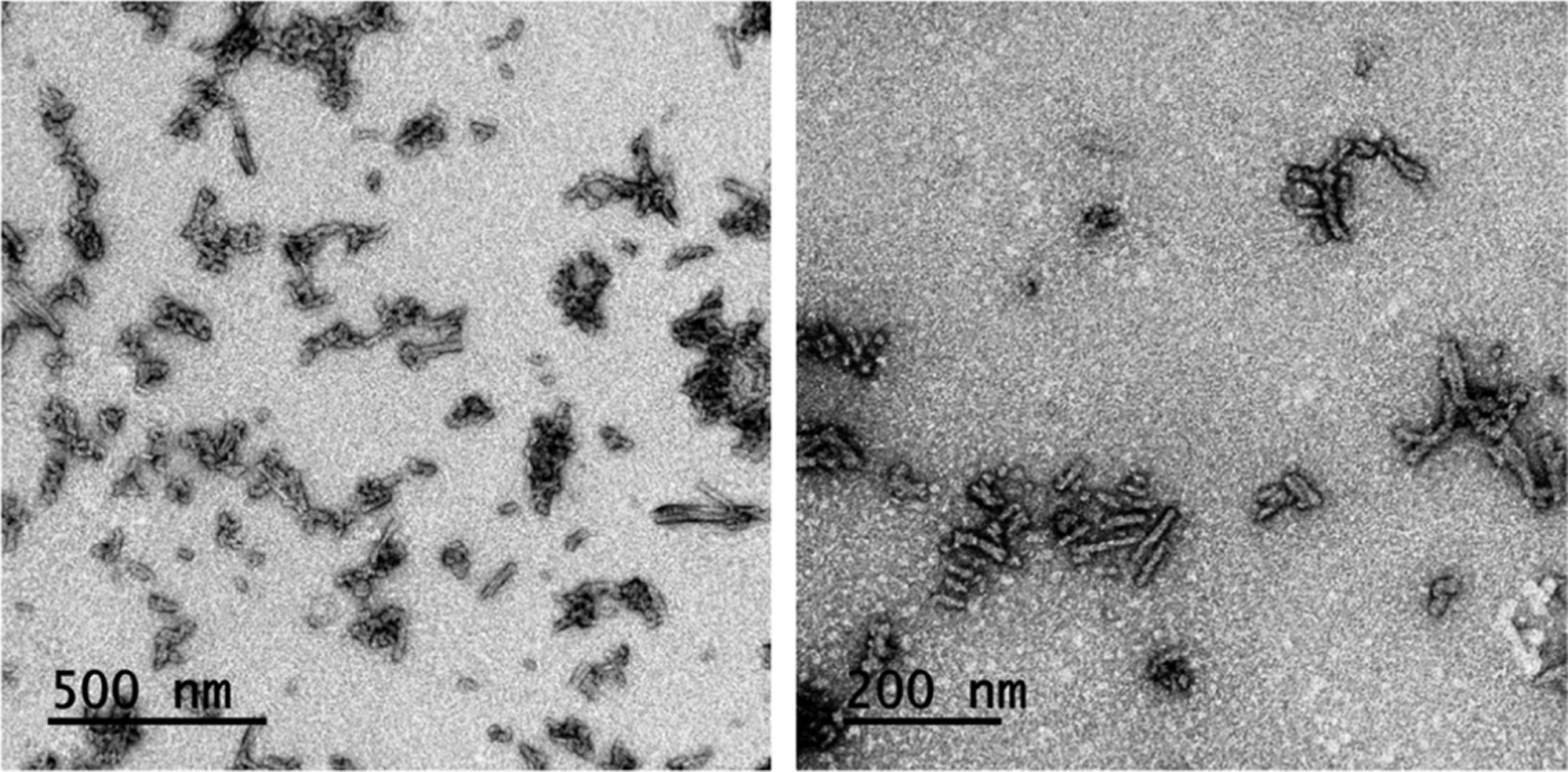
Representative TEM images of sonicated Sup35-Z amyloid fibrils upon negative staining. The scale bar corresponds to 500 and 200 nm, respectively.

The size and shape of nanomaterials impact their dispersion, cellular uptake, and delivery efficacy.^34^ We sought to generate shorter versions of our functional amyloid fibrils that can be employed as nanoparticles. To this aim, we sonicated the fibrils shortly and obtained relatively homogeneous rod-like nanostructures of 50–100 nm in length, as visualized by TEM (Figure 4). These nanorods do not spontaneously reassemble into fibrils after sonication is ceased, as corroborated by dynamic light scattering (DLS) analysis (Figure S9).

One of the main constraints for the use of amyloid-like materials in biomedicine is that they can present an inherent cytotoxicity.^35^ The toxicity is associated with oligomeric assemblies rather than mature fibrils, but it is unknown if mechanical shearing of mature fibrils might render toxic particles. To discard this possibility, the cytotoxicity of the Sup35-Z nanorods was evaluated at different concentrations, ranging from 1 to 25 μM, using the PrestoBlue assay (Figure S10). The statistical analysis using a one-way ANOVA test further corroborated that these nanoparticles did not show any significant toxicity for human HeLa cells, suggesting exceptional biocompatibility.

### Functionalized Sup35-Z Nanorods Target Human Cancer Cells Specifically

We aimed to assess if Sup35-Z nanorods can target particular epitopes in living cells once they have been decorated with specific antibodies via their Z-domains. We decorated the nanorods with either an anti-EGFR antibody or an anti-CD3 antibody, labeled with Alexa 555 and Alexa 488, respectively, as described above. Anti-EGFR antibodies target the epidermal growth factor receptor (EGFR), significantly expressed on the membrane of many epithelial cancer cells, such as HeLa cells. In contrast, anti-CD3 antibodies target the TCR/CD3 complex of T lymphocytes and consequently activate them.^36^ First of all, we incubated the anti-EGFR antibody-decorated nanorods (NRs-anti-EGFR) with HeLa cells. Most of HeLa cells were red-fluorescent when visualized by confocal microscopy, which indicated the NRs-anti-EGFR could recognize them.

**Figure 5:**
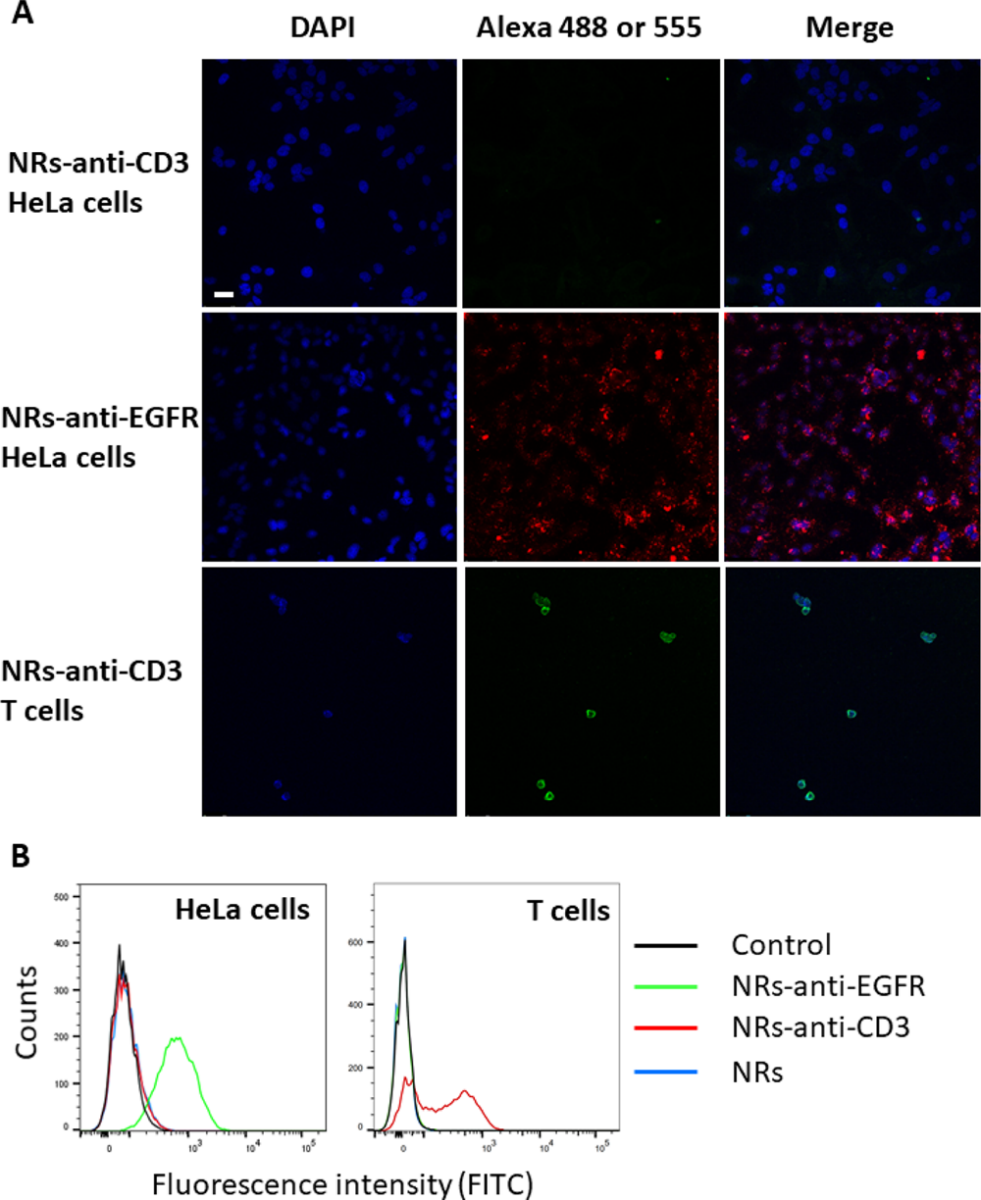
Binding selectivity of functionalized Sup35-Z nanorods to human cells. (A) Representative confocal microscopy images of HeLa cells treated with nanorods conjugated with an anti-CD3 antibody (NRs-anti-CD3, Alexa 488) (upper panel) or an anti-EGFR antibody (NRs-anti-EGFR, Alexa 555) (middle panel) and T lymphocytes incubated with nanorods conjugated with an anti-CD3 antibody (NRs-anti-CD3, Alexa 488) (lower panel). The scale bar corresponds to 50 μm. (B) Quantitative analysis of fluorescein fluorescence on HeLa cells and T lymphocytes by flow cytometry. HeLa cells or T lymphocytes were incubated with anti-EGFR-loaded Sup35-Z nanorods (NRs-anti-EGFR, green line), anti-CD3 antibody-loaded nanorods (NRs-anti-CD3, red line), and free nanorods (NRs, blue line). HeLa cells or T lymphocytes treated with PBS were used as a control. Alexa Fluor 488-labeled antibody was used as a fluorescence probe to calculate the proportion of cells bound to nanorods using a FITC detector.

In contrast, when anti-CD3 antibody-loaded Sup35-Z nanorods (NRs-anti-CD3) were incubated with HeLa cells, no cellular fluorescence was observed, as expected since this cell type does not contain the CD3 complex on its surface (Figure 5A). However, when the NRs-anti-CD3 were incubated with T lymphocytes, green fluorescent cells were visualized (Figure 5A). Thus, the data indicated that antibody-loaded nanorods’ recognition of human cells was antibody-guided and specific.

Subsequently, we loaded the nanorods with either an anti-EGFR antibody or an anti-CD3 antibody, both labeled with Alexa Fluor 488. We incubated the functionalized protein nanorods with HeLa cells or T lymphocytes, followed by a washing step. The cells were instantly analyzed by flow cytometry, monitoring the green fluorescence of Alexa Fluor 488 using a FITC emission detector. Only HeLa cells incubated with NRs-anti-EGFR and T lymphocytes treated with NRs-anti-CD3 exhibited green fluorescence (Figure 5B), whereas no fluorescence was evident for HeLa cells incubated with NRs-anti-CD3 and T lymphocytes treated with NRs-anti-EGFR. The quantitative analysis indicated that >70% of HeLa cells and >60% T lymphocytes were bound to NRs-anti-EGFR and NRs-anti-CD3, respectively, which indicates a significant binding affinity of the functionalized nanorods for the corresponding receptor-expressing cells.

To evaluate if, besides targeting specifically T lymphocytes, NRs-anti-CD3 can activate them, we carried out a T cell proliferation assay implementing a modification of the typical antibody immobilization method^36^ in which NRs-anti-CD3 acted as the antibody-immobilizing agent. The T cell proliferation response was assessed using the PrestoBlue assay. The statistical analysis with a one-way ANOVA test corroborated that NRs-anti-CD3 significantly increases T cell proliferation at a comparable level than the one resulting from incubation of cells with the immobilized anti-CD3 antibody alone. In contrast, nonantibody-loaded Sup35-Z nanorods had a negligible effect on T cell proliferation (Figure S11). Therefore, the anti-CD3 antibody in the nanorods can efficiently target and activate the T lymphocytes.

### Dual Antibody-Conjugated Sup35-Z Nanorods Direct T Lymphocytes to HeLa Cells

**Figure 6:**
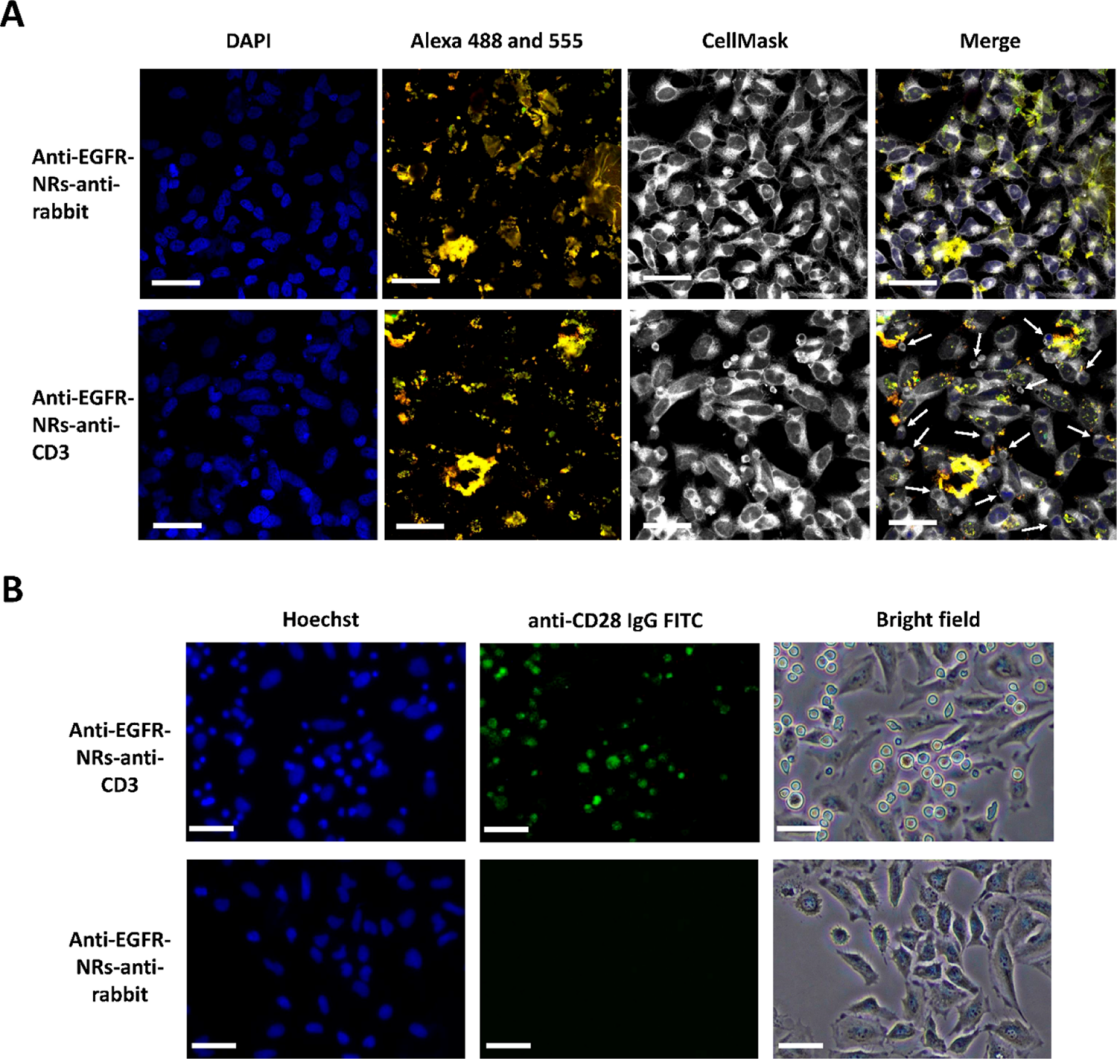
Double mAbs-conjugated nanorods redirect the CD3 expressing T cells to EGFR expressing HeLa cells. (A) Representative microscopy images of EGFR expressing HeLa cells and CD3 expressing T cells in the presence of anti-EGFR and anti-CD3-bound nanorods (anti-EGFR-Alexa Fluor 555, anti-CD3-Alexa Fluor 488, lower panel) and anti-EGFR and antirabbit-bound nanorods (anti-EGFR-Alexa Fluor 555, antirabbit-Alexa Fluor 488, upper panel), respectively. The white arrows show the presence of T lymphocytes with circular and round shapes. The anti-EGFR antibody and anti-CD3 antibody are labeled with Alexa Fluor 555 (red color) and Alexa Fluor 488 (green color), respectively. (B) Representative microscopy images of HeLa and T cells expressing EGFR and CD3 receptors, respectively, in the presence of unlabeled anti-EGFR-NRs-anti-CD3 (upper panel) and unlabeled anti-EGFR-NRs-antirabbit (lower panel) nanorods. T cells are specifically stained with an anti-CD28 IgG labeled with FITC (green fluorescence), and cell nuclei are stained with Hoechst (blue color). The scale bar represents 50 μm.

We have shown that Sup35-Z fibrils can simultaneously bind two different antibodies and that antibody-loaded nanorods can target specific cell types. This immediately suggested that they can be used to bring different cell types nearby. To confirm this idea, we loaded Sup35-Z nanorods simultaneously with two antibodies, namely, fluorescently labeled anti-EGFR and anti-CD3 (anti-EGFR-NRs-anti-CD3). We incubated the dual conjugated nanorods with HeLa cells for 20 min. Then, the medium was removed, cells were washed with PBS, and T lymphocytes were added. The mixture was incubated for 20 min, after which the medium was again removed, and cells were cleaned, mounted, and imaged. The presence of circular T cells (25 ± 5 per field) and polygonal HeLa cells connected by yellow fluorescent nanostructures (merging the anti-EGFR and anti-CD3 fluorescence channels) was observed by confocal microscopy (Figure 6A, lower panel). In contrast, Sup35-Z nanorods simultaneously loaded with anti-EGFR antibody and a fluorescent secondary antirabbit antibody (anti-EGFR-NRs-anti-rabbit) target HeLa cells but do not capture any T lymphocyte (Figure 6A, upper panel).

The presence of circular T cells nearby HeLa cells was further corroborated by specifically staining T lymphocytes with a FITC-labeled anti-CD28 antibody. As is shown in Figure 6B, anti-EGFR and anti-CD3 double-conjugated nanorods redirect green-labeled T cells to HeLa cells (upper panel). In contrast, the substitution of the anti-CD3 IgG by an antirabbit IgG precludes T cell presence (Figure 6B, lower panel). This specific dual conjugated (anti-EGFR and anti-CD3 IgGs) nanorod-triggered approximation could also be observed by staining the nucleus of HeLa cells with Hoechst and T cell membranes with wheat germ agglutinin (WGA) labeled with Alexa Fluor 555 (Figure S12).

**Figure 7:**
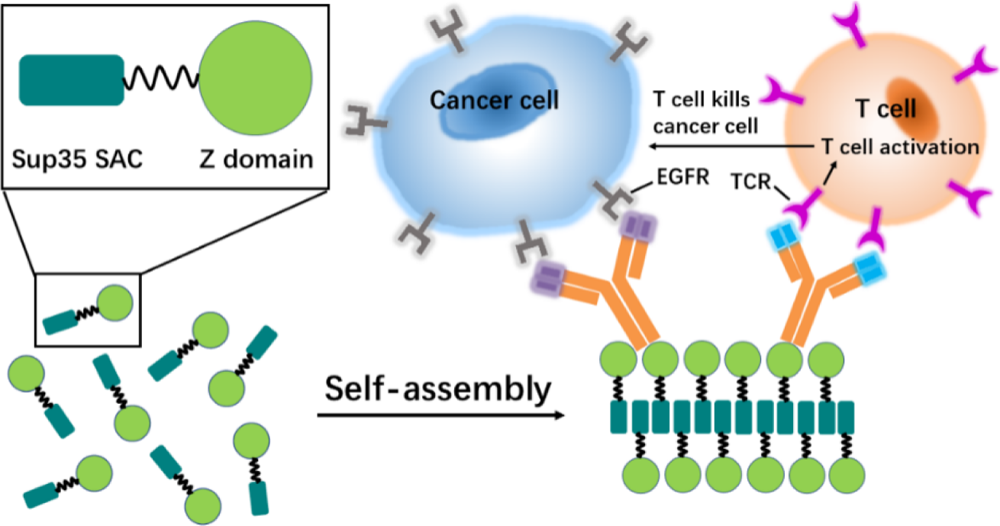
Schematic representation of the dual-targeting functionality of the mAbs-nanorod complex. The construct of Sup35-Z fusion consists of a Sup35 SAC (green square) and a Z-domain (green ball) that acts as an antibody capture domain, linked with a flexible linker (black line); Sup35-SAC induces the self-assembly of the fusion protein into antibody-binding nanofibrils. Nanorods bound to two monoclonal antibodies (mAbs) direct the TCR/CD3 complex-positive T lymphocytes to EGFR expressing tumor cells. Activated T lymphocytes would kill tumor cells.

These data indicate that dual antibody-loaded nanorods can bind at least two different antigens simultaneously and approach unrelated cell types. Thus, this nanomaterial can be potentially applied for immunotherapy as a mimetic of BsAbs (Figure 7). The combination of binding activities can be tailored at will, which together with the very large repertoire of existing antibodies anticipates that this technology might find broad application in biotechnology and biomedicine.

## Conclusions

In the present study, antibodies capturing amyloid fibrils have been built up using a hybrid protein consisting of a SAC, which provides the driving force for assembly, and the globular Z-domain, which holds a high affinity for IgGs. The fusion protein is expressed at high yield in a soluble manner, and upon assembly, the Z-domain remains in its native folded structure and keeps its functionality. We further engineered the nanofibrils’ size to obtain homogeneous nanorods that are biocompatible and can potentially have *in vivo* applications. Monospecific antibody-conjugated nanorods efficiently target the desired epitope on the surface of selected cells and, in the case of T lymphocytes, facilitate their activation.

Moreover, dual antibody-conjugated nanorods can efficiently direct T lymphocytes to HeLa cells in vitro. Thus, they act as BsAbs and hold potential for immunotherapeutic applications since they can be readily used to conjugate multiple and different antibodies and target any desired cell-type combination. They can be potentially employed for applications such as ex vivo expansion and activation of T cells in chimeric antigen receptor (CAR)-T cell therapy, using anti-CD3 and anti-CD28 antibodies,^37^ or to neutralize viruses, such as SARS-CoV-2, exploiting antibodies that target different epitopes in viral particles since multivalency is expected to increase avidity.^38^ This approach can also be exploited to target synthetic epitopes, allowing immobilization of any desired cell type to a surface of interest.

Overall, the present work illustrates a straightforward strategy for obtaining multivalent antibody-functionalized nanomaterials.

## Materials and Methods

### Reagents and Materials

Reagents were purchased from Sigma-Aldrich (UK), unless otherwise stated. Antibodies were purchased from Thermo Fisher Scientific (UK). Carbon grids (400 square mesh copper) were purchased from Micro to Nano (Netherlands), and the uranyl acetate solution was provided by the Microscopy Service (Universitat Autònoma de Barcelona). Sup35-SAC 21-residue peptides were purchased from CASLO ApS (Scion Denmark Technical University).

### Expression and Purification of Proteins

The cDNA of Sup35-Z consists of Sup35 SAC, a five-residue long linker, and Z-domain of protein A cloned in the plasmid pET28(b) with a His6 tag acquired from GenScript (USA). The construct pET28(b)/Z-domain was acquired by mutagenesis based on plasmid pET28(b)/Sup35-Z. *E. coli* BL21 (DE3) competent cells were transformed with the correspondent plasmids. Then, transformed cells were grown in 10 mL of Luria–Bertani (LB) medium containing 50 μg/mL kanamycin overnight at 37 °C and transferred into 1 L of fresh LB medium containing 50 μg/mL kanamycin. After reaching an OD_600_ of 0.6, the culture was induced with 0.4 mM IPTG at 20 °C for 16 h. Cells were collected by centrifugation at 5000 rpm for 15 min at 4 °C. The collected pellet was resuspended into 20 mL of PBS at pH 7.4 containing 20 mM imidazole, 1 mg/mL lysozyme, and 1 mM phenylmethylsulfonyl fluoride. The solution was incubated on ice, followed by sonication for 20 min. The supernatant was collected by centrifugation at 15,000 rpm for 30 min at 4 °C and purified in a His-tag column according to the manufacturer’s protocol, followed by a gel filtration onto a HiLoad Superdex 75 Prepgrade column (GE Healthcare, USA). The purified proteins were frozen with liquid nitrogen and stored at −80 °C. The purity of the sample was confirmed by SDS-PAGE. The concentration of the Z-domain and Sup35-Z proteins was determined by UV absorption using a ε value of 1490 and 5960 L·mol^–1^·cm^–1^, respectively.

### Conformational Characterization and Thermal Stability

Proteins were prepared at a final concentration of 10 μM in PBS pH 7.4 buffer. Then, samples were filtered through a 0.22 μm Millipore filter and immediately analyzed. Far-UV CD spectra were recorded from 260 to 200 nm at 1 nm bandwidth, a response time of 1 s, and a scan speed of 100 nm/min in a JASCO-815 spectropolarimeter (JASCO Corporation, Japan), thermostated at 25 °C. Ten accumulations were averaged for each spectrum. For thermal stability, ellipticity was recorded at 222 nm at each 0.5 °C with a heating rate 0.5 °C/min from 25 °C to 90 °C, using a JASCO-815 spectropolarimeter (JASCO Corporation, Japan).

### Purification of the Antibody from Bovine Serum

Soluble proteins were prepared at a final concentration of 10 μM, and a pull-down assay was performed. In particular, 50 μL of protein solution was trapped into a His-tag column equilibrated with nickel ions and then incubated with bovine serum at room temperature for 30 min. The His-tag column was washed three times with PBS buffer. Bound IgG was eluted using 0.1 M ethylenediaminetetraacetic acid, and the purity of IgG was analyzed by SDS-PAGE. For the Sup35-Z fibrils, 200 μL of the incubated protein was precipitated and washed twice with PBS buffer. The fibrils were resuspended in bovine serum and incubated at room temperature for 30 min. After that, fibrils were sedimented through centrifugation at 13,200 rpm for 20 min and washed rigorously three times with PBS buffer. Bound IgG was eluted with 0.1 M glycine-HCl pH 3.0 buffer and the purity of IgG was analyzed by SDS-PAGE.

### Aggregation Assay

Sup35-Z protein and Sup35-SAC peptides were prepared at 200 μM in PBS pH 7.4 and filtered through a 0.22 μm filter. The samples were incubated at 37 °C under agitation at 600 rpm for 5 days. The Z-domain protein incubated at the same concentrations and conditions was used as a control.

### Amyloid Dye Binding Assay

Thioflavin T (Th-T) and CR were used to determine the formation of amyloid fibrils. For the Th-T binding assay, incubated proteins were diluted to a final concentration of 20 μM in PBS pH 7.4 in the presence of 25 μM Th-T. Emission fluorescence was recorded in the 460–600 nm range, using an excitation wavelength of 445 nm and emission bandwidth of 5 nm on a JASCO FP-8200 spectrofluorometer (JASCO Corporation, Japan). For the CR binding assay, incubated proteins were prepared at a final concentration of 20 μM, and CR was mixed to a final concentration of 20 μM. Optical absorption spectra were recorded in the range from 375 to 700 nm using a Specord 200 Plus spectrophotometer (Analytik Jena, Germany). The spectra of proteins alone and buffer were acquired to subtract protein scattering.

### Transmission Electron Microscopy

For TEM sample preparation, 10 μL of the incubated proteins or incubated proteins sonicated for 5 min was deposited on a carbon-coated copper grid for 10 min and the excess liquid was removed with filter paper, followed by a negative stain with 10 μL of 2%(w/v) uranyl acetate for 1 min. Grids were exhaustively scanned using a JEM 1400 transmission electron microscope (JEOL ltd, Japan) operating at 80 kV, and images were acquired with a charge-coupled device GATAN ES1000W Erlangshen camera (Gatan Inc., USA). The width of fibrils or length of nanorods was analyzed by Image J (National Health Institute), averaging the measures of 10 individual fibrils or nanorods.

### Fourier Transform Infrared Spectroscopy

A total of 30 μL of the prepared Sup35-Z fibrils at 200 μM was centrifuged at 12,000*g* for 30 min and resuspended in 10 μL of water. Samples were placed on the ATR crystal and dried under N_2_ flow. The experiments were carried out using a Bruker TENSOR 27 FTIR spectrometer (Bruker Optics, USA) supplied with a Specac Golden Gate MKII ATR accessory. Each spectrum consists of 32 acquisitions measured at a resolution of 1 cm^–1^ using the three-term Blackman–Harris window apodization function. Data were acquired and normalized, using the OPUS MIR Tensor 27 software (Bruker Optics, USA). The IR spectrum was fitted employing a nonlinear peak-fitting equation using Origin 8.5 (OriginLab Corporation). The area for each Gaussian curve was calculated in the amide I region (1700–1600 cm^–1^) using a second derivative deconvolution method.

### Dynamic Light Scattering

The size of Sup35-Z fibrils and nanorods at different time points was determined using a Malvern Zetasizer Nano S90 (Malvern Instruments Limited, UK) in PBS buffer, pH 7.4, at 25 °C.

### Antibody Binding Activity and Binding Capacity of Fibrils

Sup35-Z fibrils were washed twice and prepared at different concentrations (0.1–0.4 μM) in PBS pH 7.4. A total of 2 μg of secondary antibody-labeled Alexa Fluor 488 was incubated with fibrils at room temperature for 30 min. For the binding assay of two antibodies, secondary antibodies labeled with Alexa Fluor 488 and Alexa Fluor 555 were mixed at ratios of 1:1, 1:2, or 2:1 and incubated with Sup35-Z fibrils at room temperature for 30 min. The fibrils were then precipitated by centrifugation at 13,200 rpm for 20 min and resuspended in PBS with washing steps. The fluorescence spectra of the original antibody and the resuspended fibrils were recorded in the range of 510 to 600 or 565 to 660 nm using an excitation wavelength of 488 or 555 nm and emission bandwidth of 5 nm on a JASCO FP-8200 spectrofluorometer (JASCO Corporation, Japan). The fluorescence intensity was calculated and fitted to a linear equation using Origin 8.5 (OriginLab Corporation). A total of 10 μL of the resuspended fibrils was added dropwise onto a clean glass slide (Deltalab, 26 × 76 mm) and covered by a cover slide (Deltalab, 22 × 22 mm). Fluorescence imaging of nanofibers was carried out on an Eclipse 90i epifluorescence optical microscope equipped with a Nikon DXM1200F (Nikon, Japan) camera and ACT-1 software. Images were acquired with an excitation filter of 465–495 nm or 540–580 nm and detecting fluorescence emission in the range 515–555 nm or 605–665 nm. Sup35 peptide fibrils were prepared at 0.4 μM and treated under the same conditions as the control.

### Functionality of Bound Antibody Displayed in Sup35-Z Fibrils

A total of 20 μL of incubated Sup35-Z protein was sedimented and washed twice with PBS buffer pH 7.4. Then, 1 μg of the mouse anti-GFP antibody was incubated with fibrils at room temperature for 30 min. The fibrils were then washed three times and further incubated with 10 μg of GFP or 2 μg of goat antimouse antibody labeled with Alexa 555. The fibrils were washed three times and resuspended in PBS buffer. A total of 10 μL of the resuspended fibrils was added dropwise onto a clean glass slide (Deltalab, 26 × 76 mm) and covered by a cover slide (Deltalab, 22 × 22 mm). The fluorescence imaging of GFP captured in the fibrils was analyzed and observed on an Eclipse 90i epifluorescence optical microscope as the previous operation. The Alexa 555 fluorescence of the secondary antibody was analyzed on a JASCO FP-8200 spectrofluorometer (JASCO Corporation, Japan), as described above. The GFP alone and secondary IgG alone were incubated with fibrils and analyzed under the same conditions as negative controls.

### Cells and Cell Culture

Human HeLa cell lines and T lymphocytes (Jurkat, clone E6-1 cell line) were obtained from American Type Culture Collection (ATCC). HeLa cells were maintained in minimum essential medium Alpha medium, supplemented with 10% fetal bovine serum (FBS). T lymphocytes were maintained in Rosewell Park Memorial Institute (RPMI 1640) medium, supplemented with 10% FBS. Both cells were incubated at 37 °C with 5% CO_2_.

### Cytotoxicity of Nanorods

HeLa cells were cultured on a 96-well plate at a concentration of 3 × 10^3^/well for 24 h. The nanorods were prepared in the range 1–25 μM and incubated with HeLa cells. Each sample was in triplicate. The plate was incubated at 37 °C with 5% CO_2_ for 48 h. PBS alone instead of fibrils was used as a control, and the medium without cells was used as a blank control. Then, 10 μL of PrestoBlue reagent (ThermoFisher Scientific) was added to each well and incubated for another 1 h. The fluorescence was analyzed on a Victor III Multilabel plate reader (PerkinElmer,USA), equipped with 530/10 nm CW-lamp filter and 590/20 nm emission filter. The viability of cells was calculated as follows
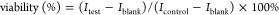
where *I*_test_, *I*_blank_, and *I*_control_ are the fluorescence intensity of test, blank, and control groups, respectively. The significance test of difference between the test group and the control was analyzed by one-way analysis of variance (ANOVA) using the Origin 8.5 program (OriginLab Corporation).

### Preparation of Antibody-Conjugated Nanorods

The incubated protein Sup35-Z was precipitated by centrifugation at 13,000 rpm for 30 min. The precipitate was sonicated for 5 min and resuspended in PBS buffer pH 7.4 containing 1 μg of antibody and incubated for 30 min. For the two antibody-conjugated nanorods, 1 μg of each antibody was used. The concentration of nanorods was determined by the reduction of absorbance at 280 nm in the supernatant fraction. The following four labeled antibodies (Thermo Fisher Scientific, USA) were used in this study: anti-EGFR antibody labeled with Alexa Fluor 488 or 555, anti-CD3 antibody labeled with Alexa Fluor 488, and goat antirabbit antibody labeled with Alexa Fluor 555. The antibody-conjugated nanorods were washed three times with PBS buffer to remove the unbound antibodies and resuspended in PBS buffer. The antibody-loaded nanorods (NRs-anti-EGFR, NRs-anti-CD3, anti-EGFR-NRs-anti-CD3, and anti-EGFR-NRs-anti-rabbit) were used for consequent experiments immediately.

### Antibody-Conjugated Sup35-Z Nanorods Target Human Cells

HeLa cells were cultured on an eight-well Millicell EZ slide (Millipore, Germany) to a final confluence of 70–80%. Then, the medium was replaced with fresh medium containing 10 μM NRs-anti-EGFR, and the slide was incubated at 37 °C, 5% CO_2_, for 20 min. The anti-CD3 antibody-loaded nanorods (NRs-anti-CD3) were used as a control. The medium was removed, and the adherent cells on the slide were rinsed three times with fresh medium. For the lymphocytes, cells were harvested and resuspended in fresh medium containing 10 μM NRs-anti-CD3. Then, cells were incubated at 37 °C, 5% CO_2_, for 20 min, followed by centrifugation and washed three times with fresh medium. A total of 150 μL of the suspension was transferred to the wells of the slide and incubated at 37 °C, 5% CO_2_, for 20 min. The medium was removed slightly, and the cells were slightly rinsed three times. After that, the treated cells were fixed with 4% paraformaldehyde (PFA) at room temperature for 20 min, followed by a washing step with PBS buffer. The four tabs were broken, and 10 μL of mounting medium containing DAPI was added dropwise onto each well of slide. A coverslip was put on the slide. The slide was observed on a Leica TCS SP5 confocal microscope (Leica Biosystems, Germany) and images were acquired by using 405, 488, and 561 nm excitation lasers for DAPI, Alexa Fluor 488 and 555, respectively.

### Flow Cytometry Assay

The NRs-anti-EGFR, NRs-anti-CD3, and NRs were prepared as described above. HeLa cells or T lymphocytes were prepared in PBS buffer pH 7.4 at a final concentration of 1 × 10^6^ cells/mL. Then, 200 μL of cells were precipitated and then resuspended in 200 μL of PBS buffer containing NRs-anti-EGFR, NRs-CD3, and NRs, respectively. After 30 min of incubation, the cells were pelleted and washed three times. Then, 200 μL of the cell suspension was analyzed using a FACSCalibur cytometry (BD Biosciences, Becton Dickinson, USA), equipped with a FITC laser. Fluorescence intensities of cell-bound nanorods were analyzed and quantified using FlowJo (BD Biosciences, USA). Cells treated with PBS were used as a control.

### Proliferation Response of T Lymphocytes in the Presence of NRs-anti-CD3

A total of 50 μL of anti-CD3 antibody alone and NRs-anti-CD3 resuspension was dispensed to each well of a 96-well plate. Each sample was triplicate. A total of 50 μL of sterile PBS and fibril resuspension were used as controls. The plate was sealed with Parafilm and incubated at 37 °C for 2 h. Then, the solution was removed and each well was rinsed three times with 200 μL of PBS to remove all unbound IgG. T lymphocytes were prepared at a final concentration of 10^6^ cells/mL; 200 μL of cell suspension was added to each well and incubated at 37 °C, 5% CO_2_, for 2 days. The medium without cells was used as the blank control. The cell proliferation was analyzed using the PrestoBlue assay, as described above. Statistical calculation was performed by using the Origin 8.5 program (OriginLab Corporation), as described above.

### Two mAbs-Conjugated Sup35-Z Nanorods Redirect Lymphocytes to HeLa Cells

HeLa cells were cultured on an eight-well Millicell EZ slide (Millipore, Germany) to a final confluence of 70–80%. Then, the medium was replaced with fresh medium containing 10 μM anti-EGFR-NRs-anti-CD3. After that, the slide was incubated at 37 °C, 5% CO_2_, for 20 min. The anti-EGFR-NRs-antirabbit was used as the control. The medium was removed, and each well was rinsed three times with PBS buffer; 150 μL of lymphocyte suspension was added and incubated for other 20 min. The medium was removed, and each well was rinsed three times with PBS buffer. Cells were stained with CellMask Deep Red for 10 min. Then, cells were fixed with 4% PFA at room temperature for 20 min, followed by a washing step with PBS buffer. The four tabs were broken, and 10 μL of mounting medium containing DAPI was added dropwise onto each well of the slide, and a coverslip was put on the slide. The slide was observed using a Leica TCS SP5 confocal microscope (Leica Biosystems, Germany). Images were acquired by using 405 nm, 488 nm or 561 nm, and 633 nm excitation lasers for DAPI, Alexa Fluor 488 or 555, and CellMask deep red, respectively.

For the staining of T cells with WGA, T lymphocytes were incubated in the presence of WGA labeled with Alexa Fluor 555 for 20 min. After that, cells were harvested by centrifugation and rinsed with fresh medium. These stained T cells were added to previously stained with Hoechst HeLa cells in the presence of nonlabeled antibody-decorated nanorods (anti-EGFR-NRs-anti-CD3 or anti-EGFR-NRs-antirabbit) and incubated at 37 °C, 5% CO_2_, for 20 min. After that, the medium was removed and the slide was rinsed three times with PBS. Cells incubated with decorated NRs were visualized using an Eclipse Ts2R-FL inverted microscope (Nikon, Japan), and images were acquired using appropriate filters and under a bright field.

For the specific staining of T cells, lymphocytes and HeLa cells were incubated in the presence of double-decorated and nonlabeled NRs (anti-EGFR-NRs-anti-CD3 or anti-EGFR-NRs-anti-rabbit) at 37 °C, 5% CO_2_, for 20 min. Then, the medium was removed, the slide was rinsed three times with PBS, and T cells were specifically stained with an anti-CD28 IgG labeled with FITC for 20 min. The cell nuclei of T cells and HeLa cells were stained with Hoechst during 5 min, and the slide was finally rinsed with PBS and observed using an Eclipse Ts2R-FL inverted microscope (Nikon, Japan). The images were acquired using appropriate filters and under a bright field.

## Supporting Information Available

Schematic representation and sequence of the Sup35-Z fusion protein, biophysical characterization of Sup35-Z, antibody binding ability of Sup35-Z, FTIR spectra of Sup35-Z fibrils, antibody binding properties and stability of Sup35-Z fibrils, size distribution and cytotoxicity of Sup35-Z nanorods, T cell proliferation assay, colocalization of HeLa and T cells, and assignment of secondary structure components of Sup35-Z from FTIR spectra (PDF)
