# Supplementary material for: Dual Antibody-Conjugated Amyloid Nanorods to Promote Selective Cell–Cell Interactions
Source: ACS Appl Mater Interfaces. 2021 Mar 24;13(13):14875–84. doi: 10.1021/acsami.0c21996 (PMC9262253; doi:10.1021/acsami.0c21996)
Supplement: Supplementary file 1 — am0c21996_si_001.pdf [file am0c21996_si_001.pdf]

Supporting information for:

Dual Antibody-Conjugated Amyloid Nanorods to Promote Selective Cell-Cell Interactions

*Weiqiang Wang[1†], Marcos Gil-Garcia[1] and Salvador Ventura[1]**

[1]Institut de Biotecnologia i de Biomedicina and Departament de Bioquímica i Biologia Molecular; Universitat Autònoma de Barcelona; 08193 Bellaterra (Barcelona), Spain.

[†]Present address: Institute of Physical Science and Information Technology, Anhui University, 230601 Hefei, China

E-mail: salvador.ventura@uab.es

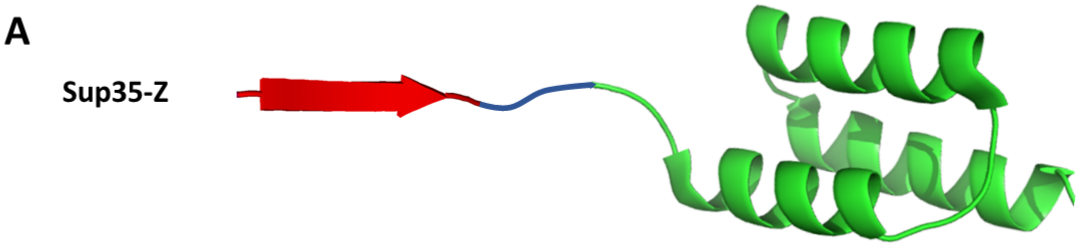

B

M G R G N Y K N F N Y N N N L Q G Y Q A G F Q S G S G S V D N K F N K E Q Q N
A F Y E I L H L P N L N E E Q R N A F I Q S L K D D P S Q S A N L L A E A K K L N D
A Q A P K H H H H H H

Figure S1. Schematic representation and sequence of the Sup35-Z fusion protein. (A) Sup35-Z with Sup35 soft amyloid core (SAC) (residues 98-118) fused to Z domain (PDB: 1Q2N), an engineered analog of the B domain of *Staphylococcus aureus* protein A is shown in cartoon representation. (B) Sequence of the Sup35-Z. The SAC, spacer linker, globular structure and His6 tag are shown in red, blue, green and black, respectively.

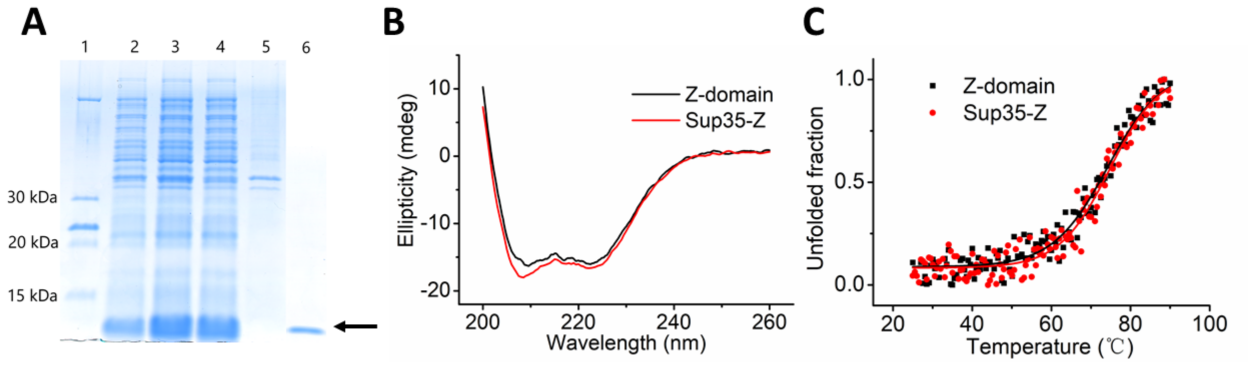

Figure S2. Solubility, conformation, and stability of Sup35-Z protein. (A) Analysis on SDS-PAGE of the expression of Sup35-Z fusion protein. *Lane 1* corresponds to molecular weight marker, *lane 2,* non-induced culture, *lane 3*, total extract induced, *lane 4*, soluble fraction (supernatant) and, *lane 5*, insoluble fraction (pellet). *Lane 6* shows the purified Sup35-Z protein by gel filtration (from another gel). A black arrow indicates the band corresponding to Sup35-Z. (B) Far-UV CD spectra of purified Sup35-Z and the Z-domain alone (Z-domain). (C) Thermal stability of both proteins analysed by far-UV CD signal at 222 nm.

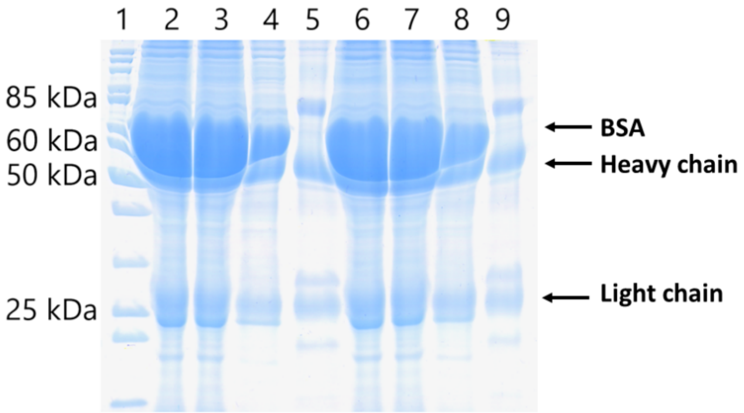

Figure S3. SDS-PAGE analysis of antibody binding affinity to soluble Z domain and Sup35-Z fusion. *Lanes 1-5*, correspond to Sup35-Z: *Lane 1* corresponds to molecular weight marker, *lane 2*, bovine serum, *lane 3*, flow-through of bovine serum after incubation with Sup35-Z domain loaded in His-tag column, *lane 4*, PBS buffer wash, *lane 5*, eluate with 0.1 M EDTA; *lanes 6-9*, correspond to the same succession of fractions for Z-domain alone loaded His-tag column.

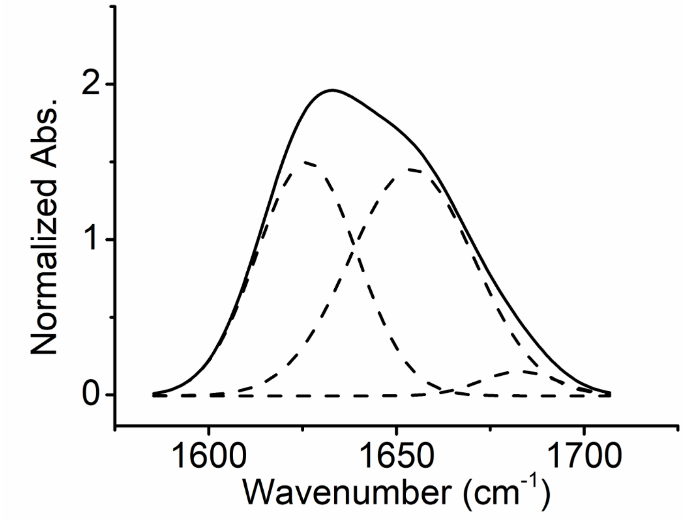

Figure S4. Conformational properties of Sup35-Z fusion protein fibrils. Sup35-Z protein solutions were incubated for 5 days. The FTIR absorbance spectra of Sup35-Z fibrils in the amide I region (solid line) and the component contributing bands (dashed lines) are shown.

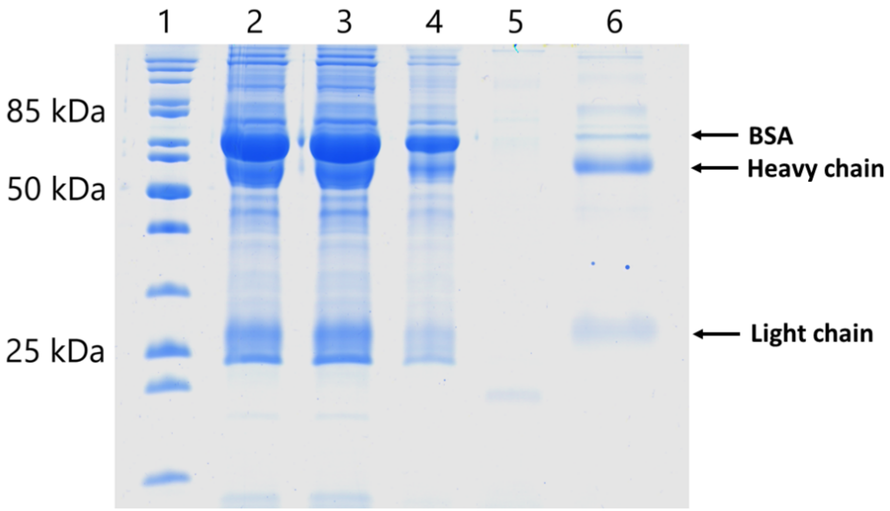

Figure S5. SDS-PAGE analysis of antibody binding affinity to Sup35-Z fusion protein fibrils. *Lane 1* corresponds to molecular weight marker, *lane 2*, bovine serum, *lane 3*, supernatant of bovine serum after incubation with Sup35-Z fibrils, *lane 4*, PBS buffer wash, *lane 5*, insoluble fraction after elution with 0.1 M glycine buffer pH 3.0. *Lane 6*, eluate with 0.1 M glycine buffer pH 3.0.

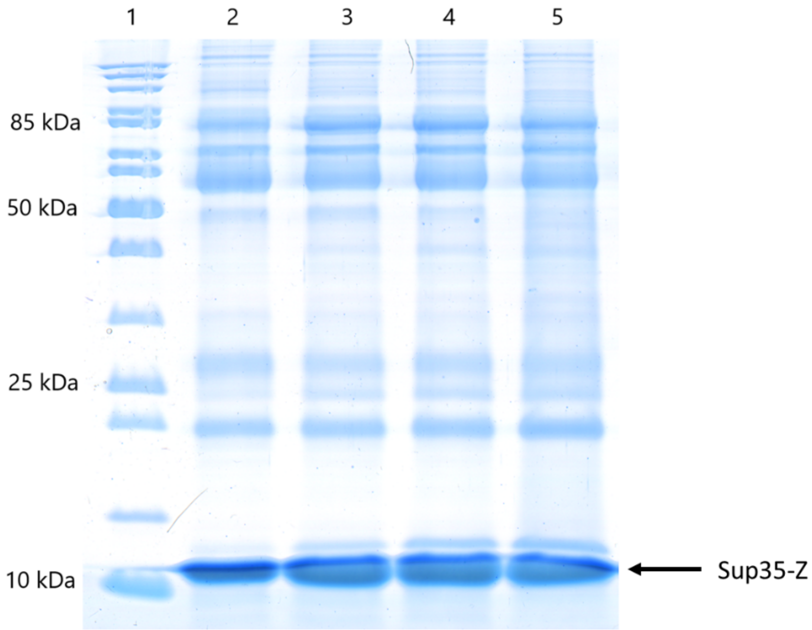

Figure S6. SDS-PAGE analysis of stability of Sup35-Z fusion protein fibrils in bovine serum. *Lane 1* corresponds to molecular weight marker, *lanes 2-5*, Sup35-Z fibrils incubated with bovine serum for 30 min, 12 h, 1 day and 3 days, respectively. The black arrow indicates the Sup35-Z fusion band.

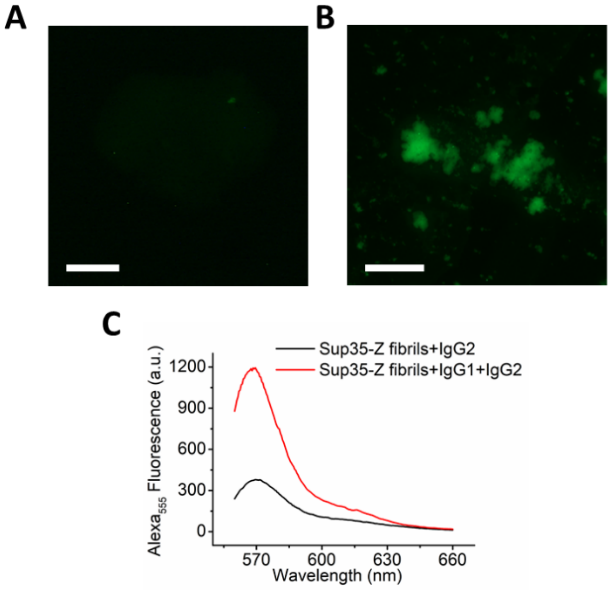

Figure S7. Functionality of the conjugated antibody on Sup35-Z fibrils. Fluorescence microscopy image of (A) Sup35-Z fibrils and (B) anti-GFP antibody conjugated Sup35-Z fibrils in the presence of GFP, scale bar represents 50 µm. (C) Fluorescence spectra of Sup35-Z fibrils incubated with an Alexa 555 labeled secondary antibody (black line) or incubated sequentially with a primary unlabeled antibody and an Alexa 555 labeled secondary antibody (red line).

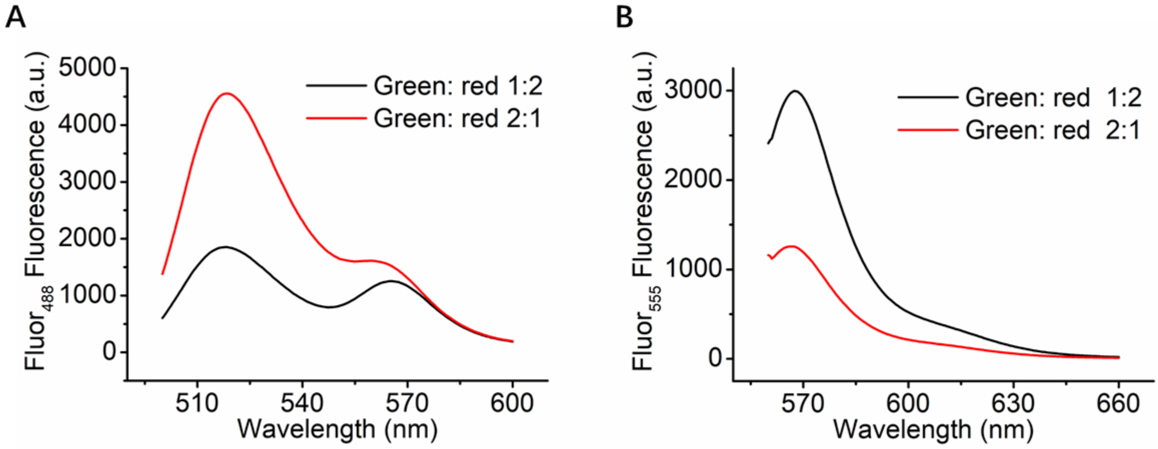

Figure S8. Fluorescence spectra of Sup35-Z fibrils incubated with antibody mixtures at desired ratios. (A) Alexa Fluor 488 fluorescence spectra of Sup35-Z fibrils incubated with a mixture of green and red labeled antibody at ratios of 1:2 and 2:1, respectively. (B) Alexa Fluor 555 fluorescence spectra of Sup35-Z fibrils incubated with mixture of green and red labeled antibody at ratios of 1:2 and 2:1, respectively.

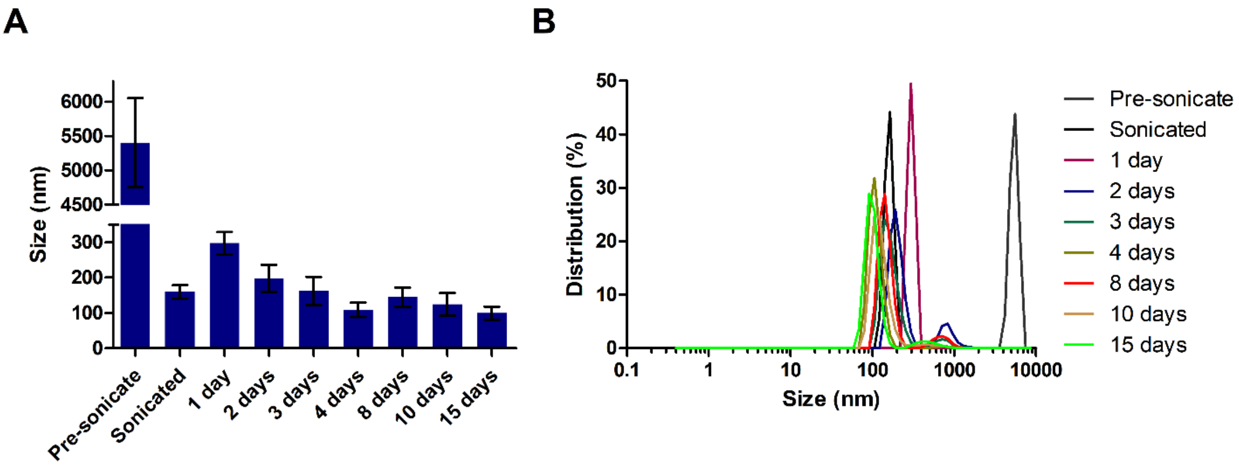

Figure S9. Dynamic light scattering (DLS) size distribution of Sup35-Z nanorods. (A) Size distribution values of Sup35-Z before and after sonication (the different days refer to the time after sonication). Values are represented as mean ± standard deviation (SD). (B) Size distribution graphics of Sup35-Z nanorods at specific time points.

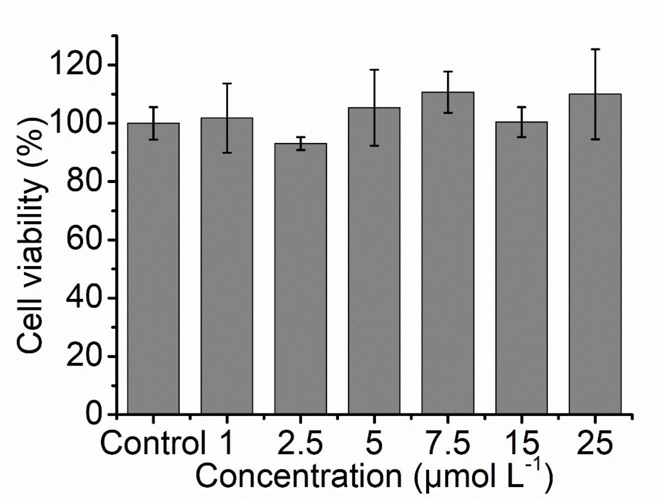

Figure S10. Cytotoxicity of Sup35-Z nanorods. Results are expressed as means ± SD, n=3, and analysed using a one-way ANOVA test. The statistical difference between the control group and the test group was established at $P < 0.05$.

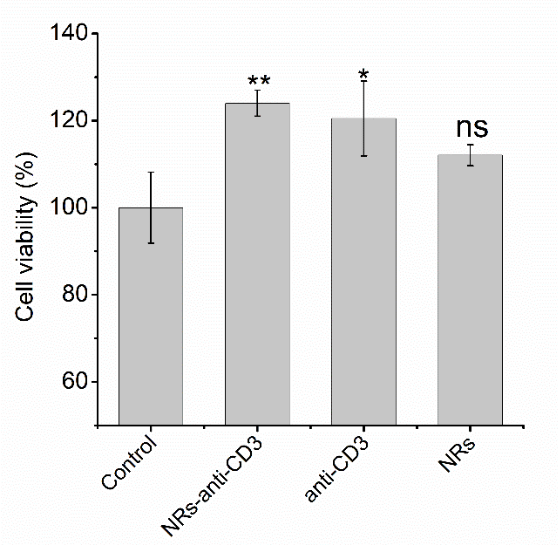

Figure S11. Proliferation response of T lymphocytes. T lymphocyte proliferation was measured in the presence of anti-CD3 antibody conjugated nanorods (NRs-anti-CD3), anti-CD3 antibody, and nanorods (NRs) alone. Results are expressed as means ± SD, n=3, and analysed using a one-way ANOVA test. The statistical differences between the control group and the test group were established at * $P < 0.05$ and ** $P < 0.01$.

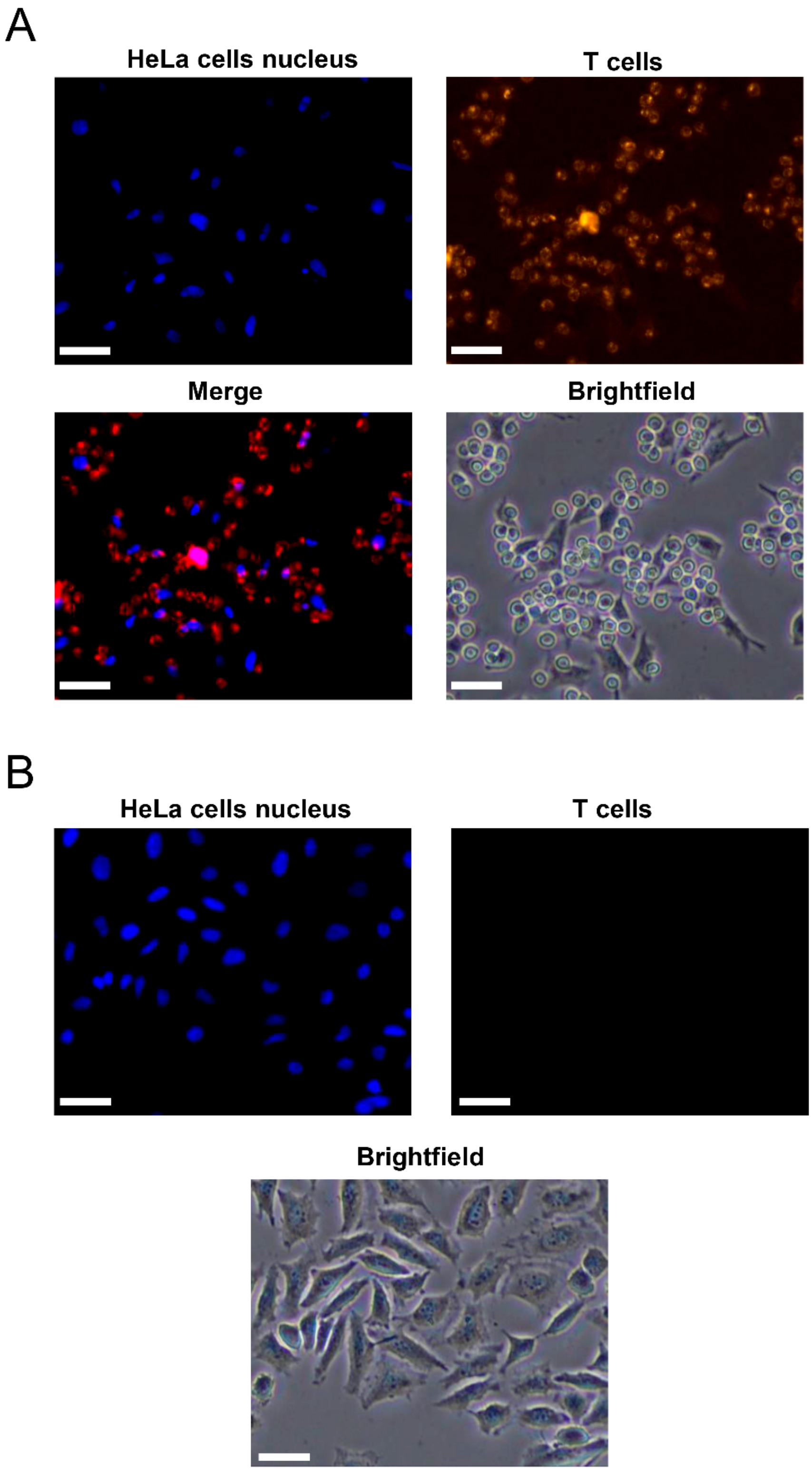

Figure S12. Double IgG-decorated nanorods direct T cells expressing CD3 to HeLa cells expressing EGFR. Representative microscopy images of EGFR expressing HeLa cells and CD3 expressing T cells in the presence of double decorated (anti-EGFR and anti-CD3 IgGs) nanorods (A) and anti-rabbit IgG bound nanorods (anti-EGFR and anti-rabbit IgGs) (B), respectively. HeLa cells nuclei were stained with Hoechst (blue color) and T cell membranes with WGA labeled with Alexa fluor 555 (red color). The scale bar represents 50 µm.

Table S1. Assignment and area of the secondary structure components of Sup35-Z fibrils in the amide I region of the FTIR spectra

**Assignments**	**Band area (%) and position**
**Inter β-sheet**	44.0 (1626 cm^{-1})
**α-helix/turns**	52.6 (1654 cm^{-1})
**β-sheet**	3.4 (1682 cm^{-1})
